# The anti-inflammatory and antifibrotic effects of *Coreopsis tinctoria* Nutt on high-glucose-fat diet and streptozotocin-induced diabetic renal damage in rats

**DOI:** 10.1186/s12906-015-0826-x

**Published:** 2015-09-07

**Authors:** Lan Yao, Linlin Li, Xinxia Li, Hui Li, Yujie Zhang, Rui Zhang, Jian Wang, Xinmin Mao

**Affiliations:** College of Traditional Chinese Medicine, Xinjiang Medical University, Xinyi Street 393, Urumuqi, 830011 China; College of Basic Medical Sciences, Xinjiang Medical University, Xinyi Street 393, Urumuqi, 830011 China; Center of Analysis and Test, Xinjiang Medical University, Xinyi Street 393, Urumuqi, 830011 China

**Keywords:** Diabetic nephropathy, *Coreopsis tinctoria* Nutt, Anti-inflammatory effect, Anti-fibrotic effect

## Abstract

**Background:**

Diabetic nephropathy is a serious complication of diabetes whose development process is associated with inflammation, renal hypertrophy, and fibrosis. *Coreopsis tinctoria* Nutt, traditionally used as a healthcare tea, has anti-inflammatory, anti-hyperlipidemia, and glycemic regulation activities. The aim of our study was to investigate the renal protective effect of ethyl acetate extract of *C. tinctoria* Nutt (AC) on high-glucose–fat diet and streptozotocin (STZ)-induced diabetic rats.

**Methods:**

A diabetic rat model was induced by high-glucose–fat diet and intraperitoneal injection of 35 mg/kg STZ. After treatment with AC at a daily dose of 150, 300 or, 600 mg/kg for 4 weeks, metabolic and renal function parameters of serum and urine were examined. Degree of renal damage, renal proinflammatory cytokines, and fibrotic protein expression were analyzed by histopathology and immunohistochemistry. Renal AMP-activated protein kinase (AMPK) and transforming growth factor (TGF)-β1/Smad signaling pathway were determined by western blotting.

**Results:**

Diabetic rats showed obvious renal dysfunction, inflammation and fibrosis. However, AC significantly reduced levels of blood glucose, total cholesterol, triglyceride, blood urea nitrogen, serum creatinine and urinary albumin, as well as expression of kidney proinflammatory cytokines of monocyte chemoattractant protein-1 and intercellular adhesion molecule-1. AC also ameliorated renal hypertrophy and fibrosis by reducing fibronectin and collagen IV and suppressing the TGF-β1/Smad signaling pathway. Meanwhile, AMPKα as a protective cytokine was markedly stimulated by AC.

**Conclusion:**

In summary, AC controls blood glucose, inhibits inflammatory and fibrotic processes, suppresses the TGF-β1/Smad signaling pathway, and activates phosphorylation of AMPKα in the kidneys, which confirms the protective effects of AC in the early stage of diabetic kidney disease.

## Background

As a result of socioeconomic development and lifestyle changes, prevalence of diabetes has risen rapidly in the past few decades. At present, diabetes has become a major public health problem in China, and solutions are urgently needed to prevent it as well as its complications [1, 2].

Diabetic nephropathy (DN) is one of the major complications of diabetes [3]. About 20 % of patients with diabetes develop nephropathy after many years [4]. DN is becoming a high-incidence cause of end-stage renal disease (ESRD) in China and other countries [5, 6]. Many studies suggest that uncontrolled hyperglycemia and hypertension may be risk factors for the development of DN [7]. Hypertrophy of glomerular structures, thickening of basement membranes, abnormal accumulation of extracellular matrix (ECM) proteins, including fibronectin and collagen, in the glomeruli, as well as persistent and slowly progressive proteinuria are the typical traits of DN [8–11].

Although much attention has been paid to strictly controlling blood glucose levels in diabetes, it is still difficult to prevent kidney disease [12, 13]. The pathogenesis of DN is complex and still not clear. However, recent experimental and clinical research has demonstrated that hyperglycemia induces renal oxidative stress, inflammation, and lipid accumulation, which leads to renal dysfunction by triggering multiple signaling pathways, and these may be crucial factors in pathogenesis of DN [4, 14, 15].

Early diagnosis and preventive treatment is of major importance for reducing morbidity and mortality of DN. Although various interventions, such as angiotensin-converting enzyme inhibitors, angiotensin receptor blockers, vitamin D receptor agonist, or antihyperlipidemic agents are widely used, the rate of side effects and cardiovascular morbidity remains high [16]. Recently, traditional herbs have been used as effective complementary therapy in DN [17].

*Coreopsis tinctoria* Nutt is a plant of the genus Asteraceae in the family of Compositae. The native species is from North America, and then it spread worldwide. A large amount of *C. tinctoria* Nutt is distributed in South Xinjiang, China, where it is known as snow chrysanthemum [18]. The dry flowers of this plant are traditionally used as a healthcare tea to prevent cardiovascular disease and diabetes. Recent research has shown that extract of *C. tinctoria* Nutt has anti-inflammatory, anti-hyperlipidemia, and glycemic regulation activities [19–21]. Since these activities are closely related to metabolic regulation and diabetic kidney protection, we tried to identify the protective effect of ethyl acetate extract of *C. tinctoria* Nutt (AC) on diabetic kidney disease, and its possible mechanism of action.

## Methods

### Preparation of AC

*C. tinctoria* Nutt was harvested from Minfeng county, Hetian city in Xinjiang province, China. The species was identified by Professor Junping Hu, College of Pharmacy, Xinjiang Medical University. The geographic coordinates of planted *C. tinctoria* Nutt was 82°, 22 ′, 00″–85°, 55′, 00 ″ of east longitude and 35°, 20 min, 00 s to 39°, 30 min, 00 s of north latitude. A voucher specimen of the plant material used in our study has been deposited in the herbarium of Ethnomedicine Research Institution in Urumuqi, Xinjiang province (No.20120715278). The dried flowers of *C. tinctoria* Nutt (160 g) were ground into powder and placed in 4 L of 55 % ethanol for reflux extraction twice at 80 °C for 2 h. The extracting liquid was filtered, merged, and then concentrated by rotary evaporator (R-210; Buchi, Essen, Germany) into 1 L liquid extract. An equal volume of AcOEt was added into the 1 L of liquid extract and than was concentrated and spray dried to obtain the powder of AC (4.4 %, w/w:dry flower). The powder of AC and metformin were weighed and dissolved in distilled water containing 1 % (g/100 mL) sodium carboxymethyl cellulose and ultrasonicated for 4 h until the powder was completely dissolved.

### Analysis of AC by HPLC/MS

AC was analyzed by HPLC (Waters 2690) with a diode-array detector (Waters 2487) scanning from 200–600 nm. Ethyl acetate extract was seperated by Shim-pack VP-ODS column (150 × 4.6 mm, 5 μm) with the optimum condition as described previously [20]. The mobile phase contained solution A of 0.5 % formic acid and solution B of acetonitrile, with a column temperature of 35 °C and flow rate of 0.3 mL/min. The elution program was as follows: 95 %–80 % of solution A and 5 %–20 % of solution B during the first 1 h; 80 %–60 % of solution A and 20 %–40 % of solution B for the remaining 50 min. A mass spectrometer (LCQ-DecA XP MAX Thermo) with a negative electrospray ionization mode was used. Mass spectrometer conditions were maitainted with a source temperature of 100 °C, desolvation temperature of 350 °C, desolvation gas flow of 600 L/h, capillary voltage of 3 kV, and cone voltage of 30 V. Mass scan range was from 100 to 800 m/z*.*

### Animal models

Healthy male Sprague–Dawley rats (8–10 weeks old, weighing 200–250 g) were obtained from the Animal Centre, Xinjinag Medical University (No. of Certification: 65000700000045). The study was authorized by the Institutional Animal Care and Use Committee of First Affiliated Hoapital of Xinjiang Medical University (Approval No.: IACUC-20140304011). The animals were kept in a specific pathogen free environment at 22 ± 2 °C and 45 ± 5 % humidity for 2 weeks of acclimatization. Then, they were randomly divided into two groups and fed with normal diet and high-fat diet (67 % normal diet, 20 % sucrose, 10 % fatty oil, 2.5 % cholesterol, and 0.8 % sodium cholate). After 8 weeks feeding, the rats were fasted overnight and injected intraperitoneally with 35 mg/kg streptozotocin (STZ) dissolved in 0.1 M citric acid and 0.1 M sodium phosphate solution, pH 4.5. The diabetic model was confirmed after 72 h by a glucose level ≥16.7 mmol/L. Animals were fed with high-fat diet until the end of the experiment.

### Experimental design

Animals were randomly divided into six groups of ten according to the weight and blood glucose levels as follows: Group 1 (NC): normal control rats with distilled water containing 1 % (g/100 mL) sodium carboxymethyl cellulose per day by gavage administration for 4 weeks; Group 2 (DC): diabetic control rats with distilled water containing 1 % (g/100 mL) sodium carboxymethyl cellulose per day by gavage administration for 4 weeks; Groups 3, 4 and 5 (DC + AC150, DC + AC300 and DC + AC600): diabetic rats with 150, 300 and 600 mg/kg AC per day, respectively, by gavage administration for 4 weeks; Group 6 (DC + M): diabetic rats with 200 mg/kg metformin per day by gavage administration for 4 weeks.

At day 27 of adminstration, 24 h urine samples from rats housed in metabolic cages were centrifuged and collected. At the end of the experiments, all the animals were killed by 10 % chloral hydrate. All the blood samples were collected. The right kidneys were rinsed with cold isotonic saline and placed immediately in liquid nitrogen, and then stored at −80 °C for testing. The left kidneys were weighed and fixed in 10 % formaldehyde solution.

### Blood sampling and analysis

The collected blood samples were centrifuged at 1000 *g* for 20 min. Serum samples were separated for detection of blood glucose, total cholesterol (TC), triglycerides, blood urea nitrogen (BUN), and serum creatinine (SCr) by an automatic biochemical analyzer (BS-120; Mindray, China).

### Urinalysis

Urine albumin levels were measured using a kit obtained from Nanjing Jiancheng Bioengineering Institute, China.

### Histopathological analysis

Left kidney tissues were fixed in 10 % formaldehyde solution, embedded in paraffin, and cut into 4 μm sections. The tissue sections were stained with hematoxylin and eosin (H&E), and Masson’s reagent. Images were captured by a light microscope (SZX7-1093; Olympus, Japan). The mesangial expansion index from 30 glomeruli of each rat was graded in four levels from 0 to 3 [22]: 0, normal glomerulus; 1, matrix expansion occurred in up to 50 % of a glomerulus; 2, matrix expansion occurred in 50 %–75 % of a glomerulus; and 3, matrix expansion occurred in 75 %–100 % of a glomerulus, and the means were caculated and compared. For Masson’s stain, 4° of fibrosis from 30 glomeruli of each rat were scored by measuring the percentage area of blue staining as follows: 0, absent or < 25 %; 1, 25 %–50 %; 2, 50 %–75 %; and 3, > 75 %. The slides from all the animals were evaluated by a pathologist who was unaware of the experimental details.

### Immunohistochemical analysis

After deparaffinization and hydration, the paraffin embedded tissue sections were washed three times with phosphate-buffered saline (PBS). Endogenous peroxidase activity was quenched by incubating the slides in methanol solution containing 3 % H_2_O_2_. After washing three times in distilled water and PBS, goat serum (Bioss Biotechnology, Beijing, China) was used to block the nonspecific binding sites for 30 min. The primary antibodies [TGF-β1, 1:500; monocyte chemoattractant protein (MCP)-1, 1:500; intercellular adhesion molecule (ICAM)-1, 1:500; fibronectin, 1:1000; Collagen IV, 1:1000; Abcam, Cambridge, MA, USA) were incubated and reacted at 4 °C overnight. On the next day, the secondary horseradish-peroxidase-conjugated antibody (Bioss Biotechnology) was added and reacted for 30 min at 37 °C. After washing three times with PBS, tissue sections were immunostained with diaminobenzidine tetrahydrochloride as the substrate and hematoxylin as the counterstain. Negative control slices were made in the same way except for the first antibody. Four degrees of TGF-β1, MCP-1, ICAM-1, Collagen IV and fibronectin expression in 30 glomeruli from each rat were graded by measuring the percentage area of positive staining as follows: 0, absent or < 25 %; 1, 25 %–50 %; 2, 50 %–75 %; and 3, > 75 %. The slides from all the animals were evaluated by a pathologist who was unaware of the experimental details.

### Western blot analysis

The right kidneys were extracted with RIPA lysis buffer (Thermo, USA) containing a protease and phosphatase inhibitor cocktail to obtain the protein lysate. The concentration of the protein lysate was measured by BCA Protein Assay Kit (Thermo). Protein lysate (20 μg) from each sample was separated by 12 % SDS-PAGE at 80 V and transferred onto PVDF membrane (Millpore, USA). After blocking in 5 % (v/v) skimmed milk and Tris-buffered saline Tween (TBST) for 1 h and washing three times in TBST, the transferred membranes were incubated with the primary antibodies of TGF-β1, β-actin (1:1000; Abcam); Smad2, p-Smad2, AMP-activated protein kinase (AMPK)α, p-AMPKα, and GAPDH (1:1000; Cell Signaling Technology, Danvers, MA, USA) at 4 °C overnight. The probed membranes were washed three times in TBST and combined with alkaline-phosphatase-conjuated secondary antibody (Invitrogen, Carlsbad, CA, USA) for 1 h at 37 °C. The protein band was colored by BCIP/NBT Substrate Kit (Invitrogen) and the band density was scanned and calculated by Quantity One v4.62 software.

### Statistical analysis

All data are presented as the mean ± SEM. Differences between multiple groups were analysed by one-way analysis of variance followed by Duncan’s multiple range test. Differences between two groups were measured by Student’s *t* test using SPSS v16.0 software (Chicago, IL, USA). Data were considerd statistically significant at *P* < 0.05.

## Results

### HPLC/MS analysis of AC

**Fig. 1:**
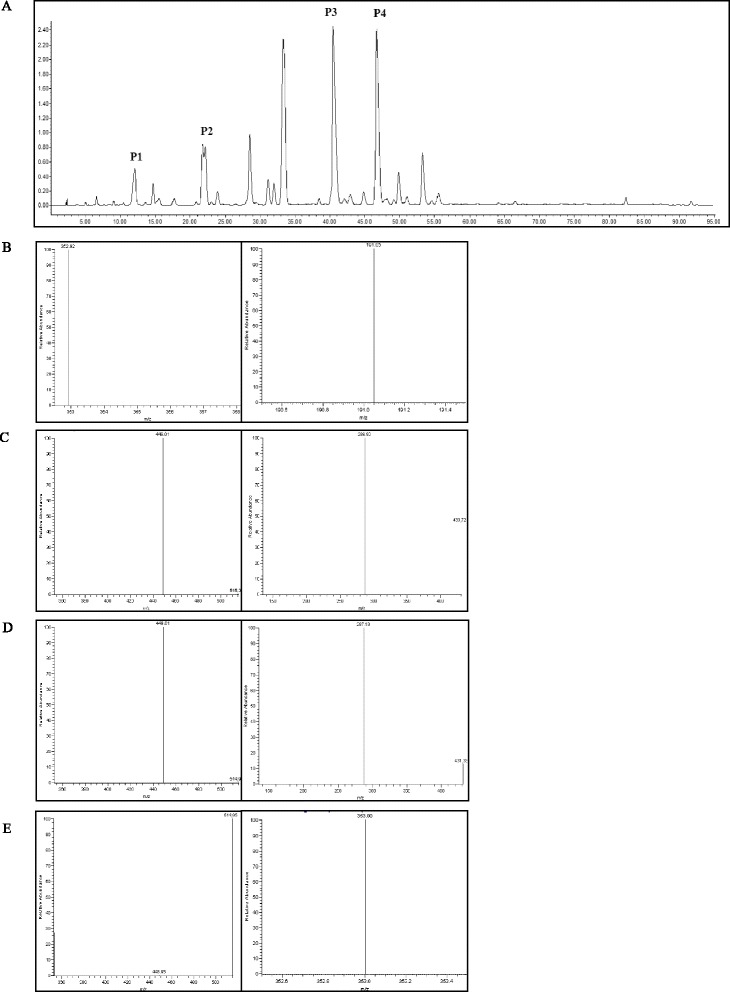
HPLC/MS analysis of AC. **a** HPLC analysis of AC at 280 nm wavelength. Four main compounds were (P1) chlorogenic acid, (P2) flavanomarein, (P3) marein, and (P4) 4,5-dicaffeoyl-quinic acid. Precursor ion (left) and product ion (right) of (**b**) chlorogenic acid, (**c**) flavanomarein, (**d**) marein, and (**e**) 4,5-dicaffeoyl-quinic acid

The main compounds and their relative content were analyzed by HPLC/MS. As shown in Fig. 1 and Table 1, we identified chlorogenic acid, flavanomarein, marien, and 4,5-dicaffeoyl-quinic acid and determined their relative content.

**Table 1:** Analysis of relative quantity and compound identification of AC

Peak no.	Compund identification	Rt (min)	Relative content (mg/g)	Precursor ion (m/z)	Product ion (m/z)	Molecular weight
P1	Chlorogenic acid	17.76	4.08	354	353	191
P2	Flavanomarein	26.3	34.73	450	449	431,287
P3	Marien	46.55	181.61	450	449	431,287
P4	4,5-dicaffeoyl-quinic acid	52.66	100.51	516	515	353

### Change in body weight and metabolic parameters after treatment with AC

Diabetes was defined as blood glucose level ≥ 16.7 mmol/L in a random blood glucose test. The DC group had a significantly higher average random blood glucose level of 30.05 mmol/L (Table 2). However, after 4 weeks of treatment, the random blood glucose level in the positive control group (*P* < 0.05) and the low-dose AC group (*P* < 0.01) was significantly decreased. By contrast, body weight and serum insulin of the DC group were significantly decreased compared with those in the normal group. Treatment with 150 mg/kg AC alleviated the body weight (*P* < 0.05) and 600 mg/kg AC (*P* < 0.05) alleviated serum insulin loss. It was noteworthy that dyslipidemia appeared in the DC, DC + AC150 and DC + AC600 groups. The later two groups were found to ameliorate abnormity of TG and TC.

**Table 2:** Changes in body weight and metabolic parametres in each group

Group	Body weigh (g)	Plasma glucose (mmol/L)	TC (mmol/L)	TG (mmol/L)	Serum insulin (μU/mL)
NC (*n =* 10)	584.80 ± 13.76	7.10 ± 0.12	1.42 ± 0.11	1.58 ± 0.07	61.23 ± 2.51
DC (*n =* 8)	344.25 ± 15.22#	30.05 ± 0.62#	7.58 ± 0.23#	23.78 ± 1.79#	26.25 ± 1.85#
DC + AC150(*n =* 10)	390.80 ± 10.88*	21.67 ± 1.79**	2.11 ± 0.33**	7.09 ± 1.17**	23.25 ± 1.47
DC + AC300(*n =* 7)	386.86 ± 17.62	30.9 ± 0.55	7.34 ± 0.26	20.27 ± 2.9	23.86 ± 1.30
DC + AC600(*n =* 9)	374.22 ± 12.25	29.1 ± 0.50	4.23 ± 0.26*	18.48 ± 1.53*	31.91 ± 1.34*
DC + M(*n =* 10)	379.1 ± 11.49	27.31 ± 0.68*	4.31 ± 0.20**	17.22 ± 0.90*	22.62 ± 1.00

Data expressed as mean ± SEM (*n =* 7–10). #*P* < 0.01 versus normal group; *P < 0.05 versus control group; **P < 0.01 versus control group

### Effect of AC on renal function

Kidney/weight ratio (K/W) of diabetic rats was markedly higher than in normal rats (Table 3). K/W only decreased significantly in the DC + AC600 group (*P* < 0.05). Twenty-four-hour urine volume was higher in diabetic than normal rats. There was no significant change in 24 h urine volume apart from in the DC + AC150 group (*P* < 0.05). In diabetic rats, mean BUN, SCr and albuminuria were notably increased in comparison with normal rats. Compared with diabetic rats, BUN level in the DC + AC300, DC + AC600 and DC + M groups was significantly decreased (*P <* 0.05). SCr level in all the treatment groups was significantly decreased, with a greater decrease in the DC + 150 group (*P* < 0.01). Albuminuria was significantly ameliorated in the DC + AC600 group (*P* < 0.01).

**Table 3:** Changes in parametres of renal function in each group

Group	K/W (mg/g)	24 h urine volume (mL/day)	BUN(mmo/L)	SCr(μmol/L)	Urine albumin (mg/24 h)
NC(*n =* 10)	2.80 ± 0.07	18.5 ± 1.85	6.26 ± 0.14	102.53 ± 0.86	4.95 ± 0.81
DC(*n =* 8)	5.15 ± 0.10#	157.88 ± 4.56#	15.96 ± 0.93#	128.56 ± 6.08#	34.59 ± 2.42#
DC + AC150(*n =* 10)	4.80 ± 0.17	133.89 ± 9.35*	13.05 ± 1.14	94.63 ± 2.20**	30.73 ± 1.53
DC + AC300(*n =* 7)	5.01 ± 0.27	142.86 ± 8.08	11.59 ± 1.5*	118.43 ± 4.98*	28.40 ± 1.44*
DC + AC600(*n =* 9)	4.71 ± 0.16*	144.50 ± 7.54	13.37 ± 0.70*	105.60 ± 4.59*	26.75 ± 2.32**
DC + M(*n =* 10)	5.03 ± 0.17	143.70 ± 5.97	12.52 ± 1.13*	103.04 ± 3.46*	27.89 ± 1.74*

Data expressed as mean ± SEM (*n =* 7–10). #*P* < 0.01 versus normal group; *P < 0.05 versus control group; **P < 0.01 versus control group

### Effect of AC on renal histology

**Fig. 2:**
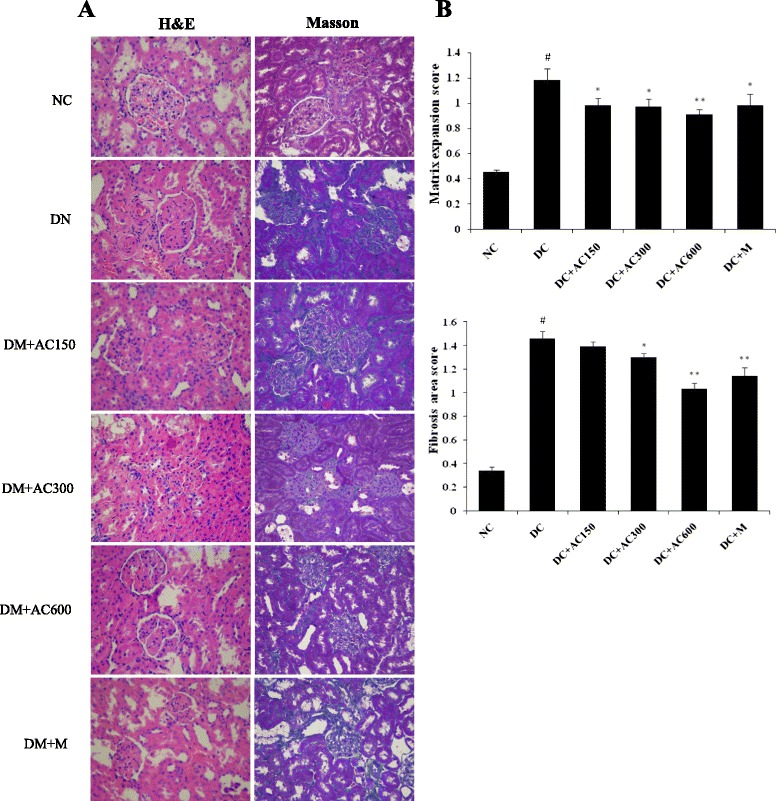
Renal histology after treatment with or without AC and metformin. **a** Pathological features of the left kidney tissue stained with H&E and Masson stain (orginal magnification 400×). Each photomicrograph represented the pathological feature of four rats from each group. **b** Matrix expansion and area of fibrosis among the groups were semiquantitatively analyzed. Values were expressed as mean ± SEM (*n =* 4). #*P* < 0.01 versus normal group; *P < 0.05 versus control group; **P < 0.01 versus control group

H&E and Masson’s stain were used to detect renal histology characteristics. Judging from H&E staining, glomerular and tubular lesions in the normal group were not obvious. The DC group showed a dramatic increase in mesangial matrix and basement membrane thickening, both in glomerular and tubular lesions (Fig. 2a). There was some improvement after treatment with 150 mg/kg AC (*P* < 0.05), 300 mg/kg AC (*P* < 0.05), 600 mg/kg AC (*P* < 0.01) and 200 mg/kg metformin (*P* < 0.05). Collagen deposition was determined by Masson’s stain. Heavy collagen deposition was clearly observed in the DC group. This abnormality was ameliorated after treatment with AC (150 mg/kg, *P* < 0.05; 300 mg/kg, *P* < 0.05; and 600 mg/kg, *P* < 0.01) and metformin (200 mg/kg, *P* < 0.01). These results confirmed the protective effect of AC and metformin on diabetic renal damage.

### Effect of AC on renal TGF-β1, MCP-1, ICAM-1, collagen IV and fibronectin expression

**Fig. 3:**
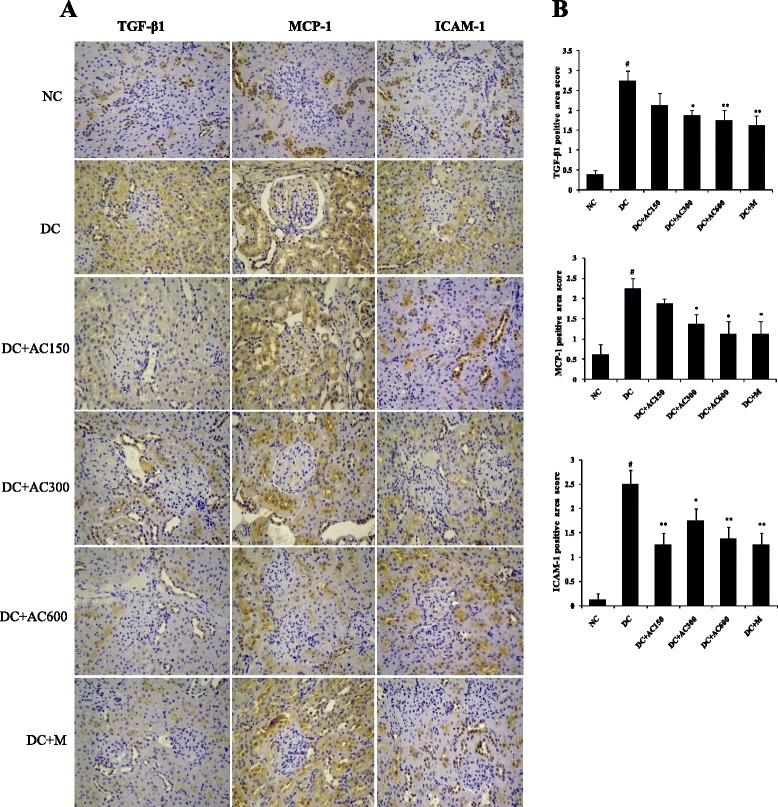
Expression of TGF-β1, MCP-1 and ICAM-1 in kidney. **a** Immunohistochemistry of TGF-β1, MCP-1 and ICAM-1 (orginal magnification 400×). **b** TGF-β1, MCP-1 and ICAM-1 expression was semiquantitatively analyzed. Values were expressed as mean ± SEM (*n =* 4). #*P* < 0.01 versus normal group; *P < 0.05 versus control group; **P < 0.01 versus control group

**Fig. 4:**
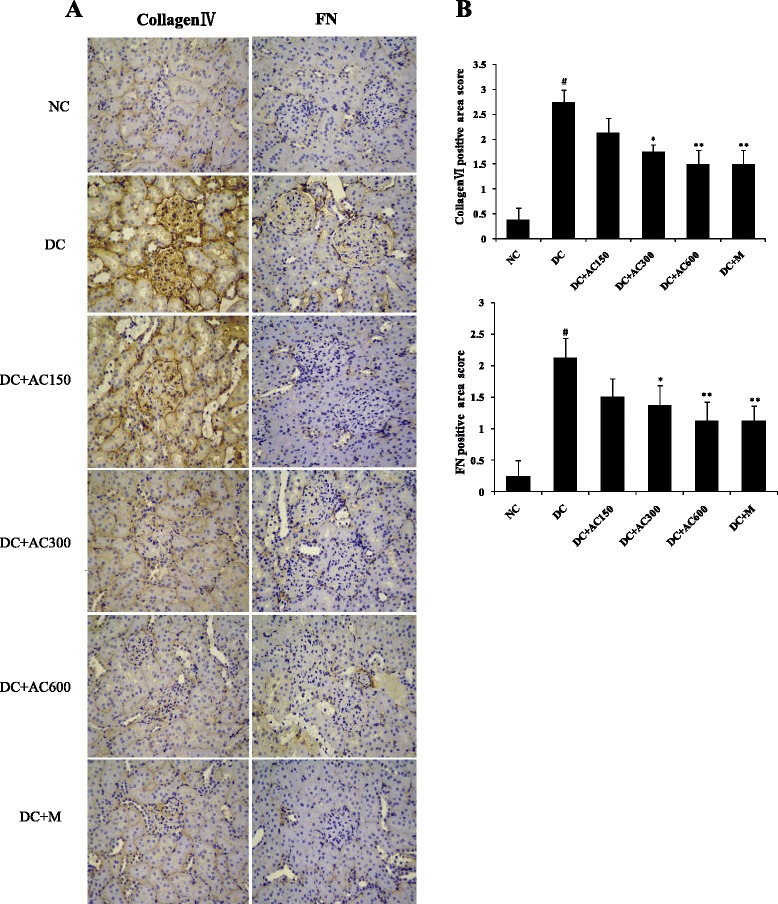
Expression of collagen IV and fibronectin in kidney. **a** Immunohistochemistry of collagen IV and fibronectin (orginal magnification 400×). **b** Collagen IV and fibronectin expression was semiquantitatively analyzed. Values were expressed as mean ± SEM (*n =* 4). #*P* < 0.01 versus normal group; *P < 0.05 versus control group; **P < 0.01 versus control group

To investigate the anti-inflammatory and anti-fibrotic effects of AC, immunohistochemistry was used to determine protein expression of TGF-β1, MCP-1, ICAM-1, collagen IV and fibronectin in diabetic kidney. Positive expression was stained brown–yellow (Figs. 3a and 4a). Compared with the NC group, the DC group showed significant overexpression of the five cytokines (Figs. 3b and 4b; *P* < 0.01). All cytokine expression was decreased to some extent after AC and metformin treatment. AC 600 mg/kg had the most inhibitory effect of protein expression of TGF-β1, MCP-1, ICAM-1, collagen IV and fibronectin. These results indicate that AC has a protective effect on diabetic renal inflammation and fibrosis.

### AC ameliorates renal inflammation and fibrosis by activing AMPK and suppressing TGF-β1/Smad signaling pathway

**Fig. 5:**
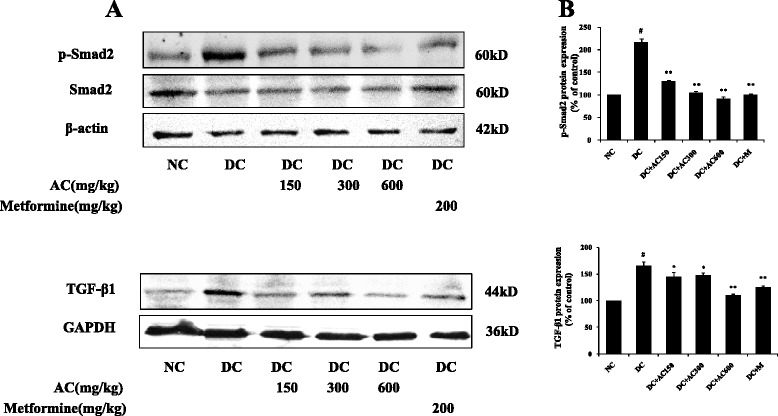
Effect of AC on protein expression of TGF-β1, Smad2 and p-Smad2. **a** TGF-β1, Smad2 and p-Smad2 expression in right kidney. **b** Band density analysis of TGF-β1, Smad2 and p-Smad2. Values were expressed as mean ± SEM (*n =* 4). #*P* < 0.01 versus normal group; *P < 0.05 versus control group; **P < 0.01 versus control group

**Fig. 6:**
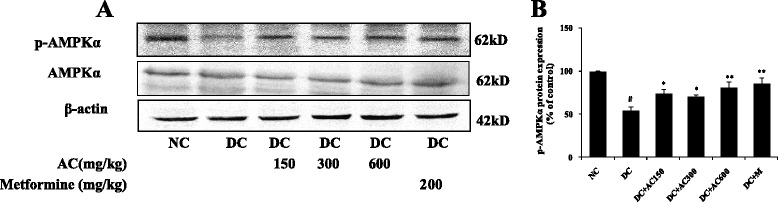
Effect of AC on protein expression of AMPK and p-AMPK. **a** AMPKand p-AMPK levels in right kidney. **b** Band density analysis of TGF-β1, AMPK and p-AMPK. Values were expressed as mean ± SEM (*n =* 3). #*P* < 0.01 versus normal group; *P < 0.05 versus control group; **P < 0.01 versus control group

To elucidate the mechanism of the anti-inflammatory and antifibrotic effects of AC, the TGF-β1/Smad and AMPK signaling pathways were investigated by western blotting. The TGF-β1/Smad signaling pathway was considered to play a vital role in progression of renal fibrosis. As shown in Fig. 5a, b, TGF-β1 expression in diabetic rats was significantly increased, followed by overexpression of phospho (p)-Smad2. Whereas, TGF-β1 and p-Smad2 expression was markedly decreased by treatment with AC. The active form of AMPKα was more highly expressed in normal rats than diabetic rats (*P* < 0.01). Treatment with AC (150, 300 and 600 mg/kg) and metformin significantly upregulated the p-AMPKα expression (Fig. 6a, b). These results indicated that the anti-inflammatory and antifibrotic effects of AC were possibly related to TGF-β1/Smad and AMPK signaling pathways.

## Discussion

In present study, we demonstrated the renal protective effect of AC in diabetic rats. After treatment with AC for 4 weeks, both glucose and lipid metabolism disorders were ameliorated. Biochemical markers of renal function, such as albuminuria, BUN, SCr, K/W ratio and 24 h urine volume, as well as renal structural abnormalities and fibrosis, were significantly improved by AC treatment. We also demonstrated that AC inhibited renal inflammatory and fibrotic reactions and upregulated p-AMPKα expression, which suggests that the positive effects of AC in diabetic nephropathy are multifactorial.

The animal model of high-fat diet combined with STZ-induced diabetes manifests many characteristics of human diabetes, such as hyperglycemia, hyperlipemia, lack of insulin secretion, and loss of body weight. Furthermore, kidney disease develops under these conditions. Compared with normal rats, diabetic rats showed significant increases in albuminuria, BUN, SCr, K/W ratio and 24 h urine volume, along with renal inflammation and renal structural abnormalities and fibrosis, which indicated that the diabetic renal injury model was successfully formed.

Previous studies that demonstrated the anti-hyperlipemia and glycemic regulation activities of C*. tinctoria* Nutt [19, 20] were confirmed by our results. After treatment with low—and high-dose AC for 4 weeks, TC and triglyceride levels were significantly decreased. Blood glucose was only decreased in the low-dose AC group, and insulin secretion was only increased in the high-dose AC and metformin groups. It was surprising that the middle dose of AC had no effect on body weight and metabolic parameters, even when it ameliorated renal effects. The low dose of AC had a better effect on lipid regulation than the high dose had, even if there was less renal damage than with high dose AC. Further research is needed to reveal the relationship bewteen different doses of AC and their renal protective effect. Renal function parameters were improved by all three doses of AC. These results indicate that the protective effect of AC on DN is not only mediated by glycemic regulation, but also by multiple synergistic effects.

Inflammation has emerged as an important factor in understanding the pathogenic mechanism of DN. Activation of the inflammatory response, leading to upregulation of various inflammatory cytokines, chemokines, growth factors, adhesion molecules, and nuclear factors, is a common pathway for progression of DN in humans and in animal models [14, 15]. MCP-1 is an important inflammatory cytokine that takes part in the development of DN. Evidence demonstrates that metabolic disorders induced by diabetes could drive MCP-1 activation and accelerate macrophage recruitment from the circulation to the kidneys. Meanwhile, high expression of MCP-1 aggravates ECM accumulation and protein excretion in glomeruli, tubules and interstitium, by activating overexpression of other proinflammatory cytokines [23, 24]. Also, MCP-1 can modulate mesangial matrix accumulation by TGF-β1 production without affecting glomerular macrophage accumulation [25]. ICAM-1 is a cell surface glycoprotein that is expressed on kidney endothelial, epithelial and mesangial cells and leukocytes. ICAM-1 promotes macrophage infiltration in glomeruli and interstitium, which is thought to play a crucial role in renal injury and proteinuria. Although the precise role of ICAM-1 in the development of DN has not been fully investigated, accumulating evidence has confirmed a close relationship. Proteinuria increases with overexpression of ICAM-1, which indicates that ICAM-1 could be a useful biomarker for prediction of DN [26]. Consistent with these studies, we found that both MCP-1 and ICAM-1 expression was markedly increased in rat diabetic kidneys, While AC treatment dramatically decreased both MCP-1 and ICAM-1 expression. These results indicate the renal improvement effect of AC is possibly mediated by its anti-inflammatory activity.

A large body of evidence shows that TGF-β1 is a key participant in the cascade of events of kidney sclerosis. Overexpression of TGF-β1 in diabetic kidney promotes cellular hypertrophy, stimulates ECM biosynthesis, and induces renal scarring in experimental and human DN [27, 28]. It is well established that TGF-β1 exerts its biological effects by activating downstream mediators of Smad2 and Smad3. The activated Smad2 and Smad3 subsequently bind to Smad4 to form the Smad complex, which translocates into the nucleus, and regulates target gene transcription, in conjunction with other nuclear cofactors [29–31]. Therefore, modulation of TGF-β1 expression could be regarded as an effective therapeutic strategy for kidney diseases [29]. We showed that rats with STZ-induced diabetes had high expression of TGF-β1, major mesangial matrix expansion, and heavy collagen deposition, as well as overexpression of fibronectin and collagen IV. These results are in accordance with a previous study that showed a close relationship between overexpression of TGF-β1 and renal morphological changes and fibrosis [28]. Diabetic rats with AC treatment showed downregulation of TGF-β1, fibronectin and collagen IV, as well as fewer renal lesions compared with untreated diabetic rats.

AMPK acts as an energy sensor in cellular responses by raising intracellular AMP and lowering ATP [32, 33]. AMPK consists of three subunits, designated α, β and γ. The α subunit of AMPK contains the catalytic domain, which has two isoforms, α1 and α2. The active form of AMPK depends on the phosphorylation of threonine-172 of the α subunit [34]. Activity of AMPK in the kidneys could be mediated by conditions of metabolic stress, such as diabetes or obesity [35]. Increasing evidence demonstrates that suppression of phosphorylation of AMPK in animal models of diabetes is involved in the pathogenesis of kidney disease through multiple pathways [36]. Recent studies have demonstrated that AMPK is closely related to renal inflammation and fibrosis [37, 38]. *In vitro* and *in vivo* experiments have confirmed that the reduction in AMPK phosphorylation could exacerbate abnormal ECM protein synthesis and renal hypertrophy by stimulating mammalian target of rapamycin activity and TGF-β1/Smad4 signaling pathway [39, 40]. Furthermore, a renal anti-inflammatory effect of active AMPK by suppression of proinflammatory cytokines such as MCP-1 has been identified [35, 39]. Consistently, AMPK agonists of 5-Aminoimidazole-4-carboxyamide.

Ribonucleoside(AICAR) and metformin inhibit diabetes-induced renal hypertrophy and inflammation response beyond their effect on hyperglycemia [36]. Therefore, AMPK could be a target for renal protection in diabetic models. Our experiments showed a marked decrease in p-AMPKα expression, along with aggravation of renal inflammation and fibrosis in diabetic rats, which was consistent with previous studies. However AC and metformin treatment significantly increased p-AMPKα expression and ameliorated renal inflammation and fibrosis. Further investigation of the effect of AC on AMPK signaling pathway is needed.

## Conclusion

Our results demonstrate that the renal protective effect of AC in rats with high-glucose–fat diet and STZ-induced diabetes is partially mediated by anti-hyperglycemic activity and partially by anti-inflammatory and antifibrotic activity via AMPK and the TGF-β1/Smad signaling pathway. However, due to the multiple compounds in AC that exert biological activities, further study is necessary to clarify the mechanism of the anti-inflammatory and antifibrotic effects of AC and its main components on DN.
